# Prenatal folic acid and vitamin B_12_ imbalance alter neuronal morphology and synaptic density in the mouse neocortex

**DOI:** 10.1038/s42003-023-05492-9

**Published:** 2023-11-08

**Authors:** Lyvin Tat, Noemi Cannizzaro, Zachary Schaaf, Shailaja Racherla, Teodoro Bottiglieri, Ralph Green, Konstantinos S. Zarbalis

**Affiliations:** 1 Department of Pathology and Laboratory Medicine, University of California, Davis, CA 95817 USA; 2 Baylor Scott & White Research Institute, Center of Metabolomics, 3434 Live Oak, Dallas, TX 75204 USA; 3 Institute for Pediatric Regenerative Medicine, Shriners Hospitals for Children, Northern California, 2425 Stockton Boulevard, Sacramento, CA 95817 USA; 4 MIND Institute, University of California, Davis, CA 95817 USA

**Keywords:** Neuronal development, Developmental neurogenesis

## Abstract

Previous reports have provided evidence that insufficient or excessive maternal folic acid (FA) intake during pregnancy can alter neurodevelopment of the offspring by modulating prenatal neurogenesis. Furthermore, our earlier work in a mouse model confirmed long-term structural changes at the cellular level of either deficient or excessive FA supply by comparably reducing dendritic arborization of cortical projection neurons. Here, we report that excessive amounts of FA decrease arborization of deep layer projection neurons, but not upper layer neurons and that reduced complexity of deep layer neurons is not observed when folic acid is replaced by folinic acid, a stable reduced form of folate. In addition, deficiency of B_12_, a vitamin that critically regulates folate metabolism, causes even more marked decreases in neuronal arborization in both deep and upper layer neurons and particularly in combination with FA excess. Furthermore, both FA excess and B_12_ deficiency affect synaptic density and morphology. Our findings point to neurodevelopmental risks associated with insufficient amounts of prenatal B_12_, particularly in association with high levels of FA intake, suggesting that the neurodevelopmental program is sensitive to an imbalance in the status of these interacting micronutrients.

## Abstract (web summary)

Prenatal folic acid excess and vitamin B12 deficiency are shown to adversely influence neurodevelopment in mice by delaying the generation and migration of late born cerebral cortical neurons and diminishing neuronal dendritic arborization.

## Introduction

Data collected by the Centers for Disease Control and Prevention (CDC) suggest that in recent decades several neurodevelopmental disorders have seen considerable rises in prevalence, chief amongst them autism spectrum disorder (ASD)^1^, but also attention deficit hyperactivity disorder (ADHD)^2^, and epilepsy^3^. For instance, the CDC reports in their latest assessment that one of every 44 children in the U.S. will be diagnosed with a disorder on the spectrum, representing an ~227 fold increase from the 1970s when prevalence was assessed at 1 in 10,000 children^4^. While it is possible that autism diagnosis has become more sensitive or expanded to include previously otherwise categorized disorders in children, the prevailing view suggests that ASD rates have substantially increased over recent decades^5^. Furthermore, this rapid rise in autism rates strongly implicates environmental rather than genetic mechanisms, as mutation rates in populations are not known to rise as quickly^6^. Indeed, an array of environmental factors, including toxins and pollutants have been proposed as causative or contributory and in some instances, such as maternal inflammation or use of anticonvulsants during pregnancy, have been validated^7^.

One environmental exposure that has increased substantially over recent decades is intake of the B vitamin folate in the synthesized, oxidized form of folic acid (FA, B_9_). Folate, is an essential micronutrient, as humans are unable to produce it de novo and require dietary intake to meet metabolic needs^8^. In its natural form, folate is found in higher concentrations in leafy greens, fruits, and legumes. Reduced forms of folate are obligatory for metabolic processes that include nucleotide synthesis, required for cellular proliferation, or supply of methyl groups, needed for methylation reactions including those essential for epigenetic control. Due to these specific metabolic roles, the recommended daily intake for folate in women during pregnancy to support the growing embryo/fetus is 1.5-fold greater than for non-pregnant women. Since recognition of the connection between inadequate folate supply and neural tube defects^9–11^, FA has been added in many countries to a staple in the diet through mandatory fortification programs, usually of cereals or grains. In addition, FA is present in prenatal supplements and has also been used to further fortify breakfast cereals and other snacks which are promoted for their health benefits as medical foods. As a consequence total folate intake, largely in the form of FA has risen substantially as have blood folate levels in populations, with a considerable proportion persistently exhibiting extreme supraphysiological concentrations in their blood^12–14^, as well as increases in unmetabolized folic acid (UMFA) in those taking supplements^14,15^.

Epidemiological research attempting to uncover associations between FA supplementation and ASD rates has produced conflicting results with the prevailing view suggesting a protective effect^16–20^. Dissenting studies, however, either did not find any association^21,22^ or concerningly, suggest a positive association between high amounts of FA intake and ASD prevalence^23–26^. Beyond ASDs, the Spanish INMA study found that high amounts of supplemental FA (≥1000 μg/d) during pregnancy was associated with impaired neurocognitive development in children at 4–5 years of age^27^ mirroring, to some extent their finding that low doses of FA (≤400 μg/d), was also associated with attentional dysfunction in boys^28^. In addition, very high FA supplement doses during pregnancy of more than 5000 μg/d lead to significantly decreased psychomotor scale scores in children compared with children whose mothers used a recommended dosage of FA supplements (400–1000 μg/d)^29^.

The folate cycle is critically dependent on the availability of the essential micronutrient, vitamin B_12_ (B_12_), which is a required cofactor for the methionine synthase reaction in which homocysteine is converted to methionine through transfer of a methyl group from N-5-methyltetrahydrofolate (CH_3_-THF). B_12_ is needed for this reaction to enable folate cycle progression and regeneration of tetrahydrofolate (THF) from CH_3_-THF. In the absence of B_12_, folate becomes functionally trapped in the form of CH_3_-THF. This suggests that the effects of FA excess, which paradoxically may decrease functional folate availability, can be further exacerbated by B_12_ deficiency, as studies on cognitive performance of older adults have suggested^30^. Irrespective of folate status, B_12_ deficiency has been recognized as a neurodevelopmental risk factor^31,32^. Furthermore, maternal B_12_ status is associated with infant B_12_ and low serum B_12_ is linked to impaired neurocognitive development in infants and young children, as documented in multiple recent reports^33–35^.

In our earlier work, we provided evidence that in mice high amounts of maternal FA intake during pregnancy (10 × the daily requirement) can alter prenatal neurogenesis and decrease neuronal arborization in the offspring^36^. Moreover, with respect to neurodevelopmental outcomes remarkably, FA excess appeared to closely mimic FA deficiency, a recognized cause of neurodevelopmental disorders. Here, in an extension of our previous work^36^, we report that offspring to dams fed a diet of moderate maternal FA excess (5-fold higher than standard mouse chow) or fed a B_12_ deficient diet, while displaying minor effects on cortical structure, show significantly decreased neuronal complexity but increased synaptic density. The strongest effects on neuronal complexity are observed when high maternal FA is combined with B_12_ deficiency. Notably, providing dams with high amounts of folinic acid (5-FTHF), a reduced, physiological form of folate, does not produce the neurodevelopmental alterations observed when stoichiometrically equal amounts of FA are being provided.

## Methods

### Animal husbandry and diets

Mice were housed in facilities approved by the Association for Assessment and Accreditation of Laboratory Animal Care International. All animals were handled in accordance with protocols approved by the University of California at Davis’ Institutional Animal Care and Use Committee. We have complied with all relevant ethical regulations for animal testing. Dietary test groups were created by supplying C57BL/6NJ dams two weeks before being caged with a male and throughout pregnancy with Clifford/Koury-based L-amino acid defined rodent diets^37^ (Dyets Inc., Bethlehem, PA) containing specific amounts of FA/B_12_ or 5-FTHF. Breeding pairs were kept on their respective folate/B_12_ controlled diets until embryo or pup collection. Test groups included (1) 2 mg/kg FA; 0.05 mg/kg B_12_ (control), (2) 10 mg/kg FA; 0.05 mg/kg B_12_ (high FA), (3) 2 mg/kg FA; 0 mg/kg B_12_ (low B_12_), (4) 10 mg/kg FA; 0 mg/kg B_12_ (high FA, low B_12_), and (5) 11.7 mg/kg folinic acid (Aprofol AG, Basel, Switzerland); 0.05 mg/kg B_12_, (high 5-FTHF) all without succinyl sulfathiazole. The control diet containing 2 mg FA/kg chow (normal replete) provides the experimentally determined folate daily requirement (DR) for laboratory rodents^38^ endorsed by the American Institute of Nutrition for rodents^39^, corresponding to the human recommended dietary allowance of 400 μg/d.

### Tissue collection and histological analysis

Pups were collected at birth (P0) and 21 days after birth (P21). They were perfused transcardially at a speed of 1 ml/min (P0) and 2 ml/min (P21) with phosphate-buffered saline (PBS) for 10 min, followed by 4% paraformaldehyde (PFA) in PBS for 5 min. Pups were decapitated and heads further fixed in 4% PFA/PBS overnight. The following day, heads were washed with PBS for 1 h, brains removed from skulls, first transferred to 15% sucrose for 12 h at 4 °C, then to 30% sucrose for another 12 h at 4 **°**C, and finally to OCT compound (Fisher Healthcare, Hampton, NH) for 45 min at room temperature (RT) to be cryoprotectively frozen. Freezing was carried out by submerging in OCT-filled polyethylene molds set in dry ice-chilled methanol. Embedded brains were stored at −80 **°**C until use and sectioned on a Leica CM3050S cryostat, collecting 14 μm sections at every 100 μm on Superfrost Plus glass slides (Thermofisher Scientific, Waltham, MA). Sections were left to dry on a Premiere Slide Warmer XH-2004 overnight and, the following day, kept at −80 **°**C until ready to be further processed. Embryo collection took place at E14.5. After transcardial perfusion, embryonic brains were collected and fixed in 4% PFA/PBS for 3 h. Subsequently, brains were washed in PBS for 1 h and went through the cryoprotective steps, embedding, and freezing procedures outlined above for pups.

### Immunofluorescence

All immunofluorescence was carried out on slide-mounted sections. In brief, sections were lightly fixed for 10 min at RT in 4% PFA/PBS, then washed in PBS three times for 5 min each. Heat-induced epitope retrieval was performed using a Biocare Medical Decloaking Chamber NxGen using the DIVA Decloaker solution (Biocare Medical, Pacheco, CA) diluted at 1x working concentration in PBS. Samples were washed in PBS and incubated with 10% donkey serum in PBS + 0.1% Triton X-100 to permeabilize the tissue and prevent non-specific antibody binding. Primary antibodies were applied and incubated overnight at 4 **°**C while secondary antibodies applied for 2 h. at room temperature. For cleaved CASP3 immunostaining, after BRN2 and TBR1 primary and secondary antibody incubation an additional blocking step using rabbit IgG (Cell Signaling # 3900) at 1:20 dilution was performed for 1 h at RT. After washing in PBS cleaved CASP3 antibody was applied overnight at 4 **°**C. The following primary antibodies and respective dilutions were used: BRN2 (Novus Biologicals, NBP2-21585) 1:400; CTIP2 (Abcam, ab18465), 1:400; Cleaved CASP3 (Cell Signaling # 8172) 1:50; TBR1 (Abcam, ab31940), 1:400.

### TUNEL assay

Slide-mounted sections were processed with the DeadEnd Fluorometric TUNEL System (Promega, Madison, WI) following the manufacturer’s instructions. First, sections were fixed in 4% PFA/PBS for 30 min and then permeabilized with 2 μg/ml solution of proteinase K for 10 min following postfixation in 4% PFA/PBS for 15 min. Afterwards, sections were equilibrated with the proprietary equilibration buffer, labeled with a nick-end-specific fluorophore, counterstained with DAPI, coverslipped, confocally imaged, and numbers of TUNEL^+^ cells per cortical hemisphere counted.

### Golgi staining

From each group, three-week-old mice born to different dams were perfused transcardially with 20 ml of PBS at 1 ml/min. Mouse brains were collected, and tissue impregnation was performed by using a modified Golgi-Cox method according to the FD Rapid GolgiStain kit (FD Neurotechnologies, Columbia, MD). Following impregnation, 100 μm coronal sections were cut on the cryostat and mounted on Superfrost Plus slides for staining with silver nitrate solution. After staining was completed, sections were dehydrated in ethanol and mounted with Permount mounting medium (Thermofisher Scientific, Waltham, MA). Brightfield photomicrographs were acquired using an Olympus BX61 microscope with associated software at 40x magnification. To maintain best possible parallel orientation of dendritic trees to the cutting plane, brain sections for analysis were selected from bregma −1.5 mm to −2.7 mm. After recording total cortical length (0% being the cortical midline), deep and upper layer projection neurons were sampled within 500 μm of the dorsomedial 20% position. Six deep layer and six upper layer cells per group were processed for dendritic analysis by acquiring 30–70 μm stacks of 0.34 μm consecutive images. To improve sampling consistency, dendritic length was limited to no less than 130 μm. After importing images to the Fiji software platform, neurites were traced following their position along the z-axis for optimal accuracy using the Simple Neurite Tracer plugin. Reconstruction of somata and dendrites was performed using the ROI manager tool. Arborization analysis was then performed using the Sholl Analysis plugin applying a modified Sholl method for best polynomial fit^40^. First and subsequent shells were set at a radius of 5 μm and intersections at each Sholl radius were determined. Raw data were compiled and imported onto GraphPad Prism for statistical analysis by mixed-model two-way ANOVA followed by post hoc analysis using Tukey’s multiple comparison test. Changes in dendritic arbor complexity prompted assessment of dendritic spine density and morphology, where we distinguished between filopodia, thin, stubby, and mushroom spine morphology, following established criteria^41^.

### Imaging

All light microscopy was conducted on an Olympus BX61 microscope and images acquired by Olympus DP71 camera and associated cellSens software (Olympus Corporation, Tokyo, Japan). All fluorescent imaging was concluded within one week from secondary antibody application to minimize fluorescent signal degradation. Images were taken at 20× magnification using a Nikon A1 confocal microscope and associated NIS Elements software (Nikon Instruments Inc., Melville, NY).

### Metabolite analysis

The concentration of 5-MTHF and THF in brain tissue were measured by LC-MS/MS as previously described^42,43^ Samples were prepared to measure monoglutamate forms of folate intermediates following a deconjugation protocol as previously described^42^. Filtered sample extracts were injected into an Acquity UPLC system coupled to a Xevo TQ MS spectrometer (Waters Corporation, Milford, MA, USA).

Methionine, S-adenosylmethionine (SAM), S-adenosyl homocysteine (SAH), cystathionine, betaine, and choline were measured in brain tissue by LC-MS/MS as previously described^43,44^. Brain tissue was deproteinized with 4 volumes of 0.4 M perchloric acid and further diluted 1:10 with isotope internal standards in aqueous solution. After centrifugation (14,000 rpm at 4 °C for 10 min) supernatant tissue extracts (10 µl) were injected into a Nexera LC system (Shimadzu Corporation, Kyoto, Japan) coupled to a 5500 QTrap mass spectrometer (SCIEX, Framingham, MA). Peak detection and quantitation were performed using Analyst 1.7.1 (SCIEX, Framingham, MA). Two levels of quality control samples were used to monitor within and between day precision of the method. The coefficient of variation (cv) was less than 15% for all metabolites.

Brain tissue MMA was determined by LC-MS/MS as described previously, with some modification^45^. Brain tissue samples were prepared as follows: Brain tissue was deproteinized in 4 volumes of 0.1 M PCA and centrifuged. A calibration stock solution of MMA (1 mM) was diluted in type 1 water to perform a 5-point calibration curve (1.25–20 µM). PCA extracts were prepared by the addition of 80 µl of water containing 0.1% formic and 5 µM d3-MMA internal standard to 20 µl of blank, standard, QC, or brain extract and mixed by vortex. The prepared sample was transferred to a 96-well microtiter plate, and 10 µl was injected for analysis into a Nexera LC system (Shimadzu, Kyoto, Japan) interfaced with a 5500 QTRAP (SCIEX, Framingham, MA, USA).

### Statistics and reproducibility

All data were obtained from at least five pups (male and female) of at least two separate litters from each nutritional group. As no significant differences between sexes were observed, data are reported combined for both sexes.

For histological data, counts were performed in NIS elements by first measuring cortical length and adjusting endpoints according to developmental stage as outlined below. In P0 pups, the ventricular apex was chosen as a starting point (0% position) to measure neocortical length. From this point, a curved line following the ventricle was drawn to the basolateral end of CTIP2-labeled layer V (100% position). After having established neocortical length, the 20% distance from the dorsal endpoint was measured and within a 200 μm-wide box around this point cells were counted. In P21 pups, the cortical midline was selected as starting point, and then by following the white matter a line drawn until the end of layer V, as determined by CTIP2 labeling. After identifying the 20% position, a 500 μm-wide box was drawn around this point within which cells were counted. All counts were performed by two observers on two neighboring sections of each brain and the numbers averaged to a single data point depicted in the bar diagrams.

All statistical analyses were performed with GraphPad Prism 9. Subsequently, variance, standard deviation, and normality (Shapiro–Wilk test) was determined when appropriate to analyze datasets. Statistical tests were done by one-way ANOVA followed by Dunnett’s multiple comparison *post hoc* test comparing all groups with the control group. TUNEL and CASP3 analyses were performed by *t*-test. Sholl analyses were conducted by a mixed-model repeated measures two-way ANOVA using a compound symmetry covariance matrix and fitted using Restricted Maximum Likelihood to handle missing values that inevitably occur in dendritic arbor analyses of neurons of varying lengths. Thus, analyses can be interpreted like a repeated measures ANOVA with missing values. Dendritic spine analysis was performed by two-way ANOVA followed by Dunnett’s multiple comparison *post hoc* test. Results were statistically significant if *p* ≤ 0.05. In the figures, bar diagrams depict the mean and standard error of the mean. Similarly, in the text mean ± standard error of the mean is provided. Individual data points correspond to biological replicates. The extent of significance between groups is indicated with one, two, three, or four asterisks if *p* values were equal to or less than 0.05, 0.01, 0.001, or 0.0001 respectively.

### Reporting summary

Further information on research design is available in the Nature Portfolio Reporting Summary linked to this article.

## Results

### FA excess or B_12_ deficiency delays the generation and/or migration of cortical upper layer projections neurons

To test the effects of gestational maternal folate/B_12_ intake on the offspring, we created five nutritional groups of C57BL/6NJ dams that were fed amino acid defined rodent chow (Dyets Inc., Bethlehem, PA): (1) control, (2) high FA, (3) low B_12_, (4) high FA/low B_12_, and (5) high 5-FTHF. Diets were initiated two weeks prior to breeding and maintained throughout pregnancy and up to weaning at three weeks of age. Pups were collected on postnatal days (P) 0 and P21 and brains processed for histological and biochemical analyses. We focused our investigation on the cerebral cortex, the predominant site of higher-order functions, such as cognitive and emotional processing and central to a multitude of neurodevelopmental disorders^46–48^.

**Fig. 1:**
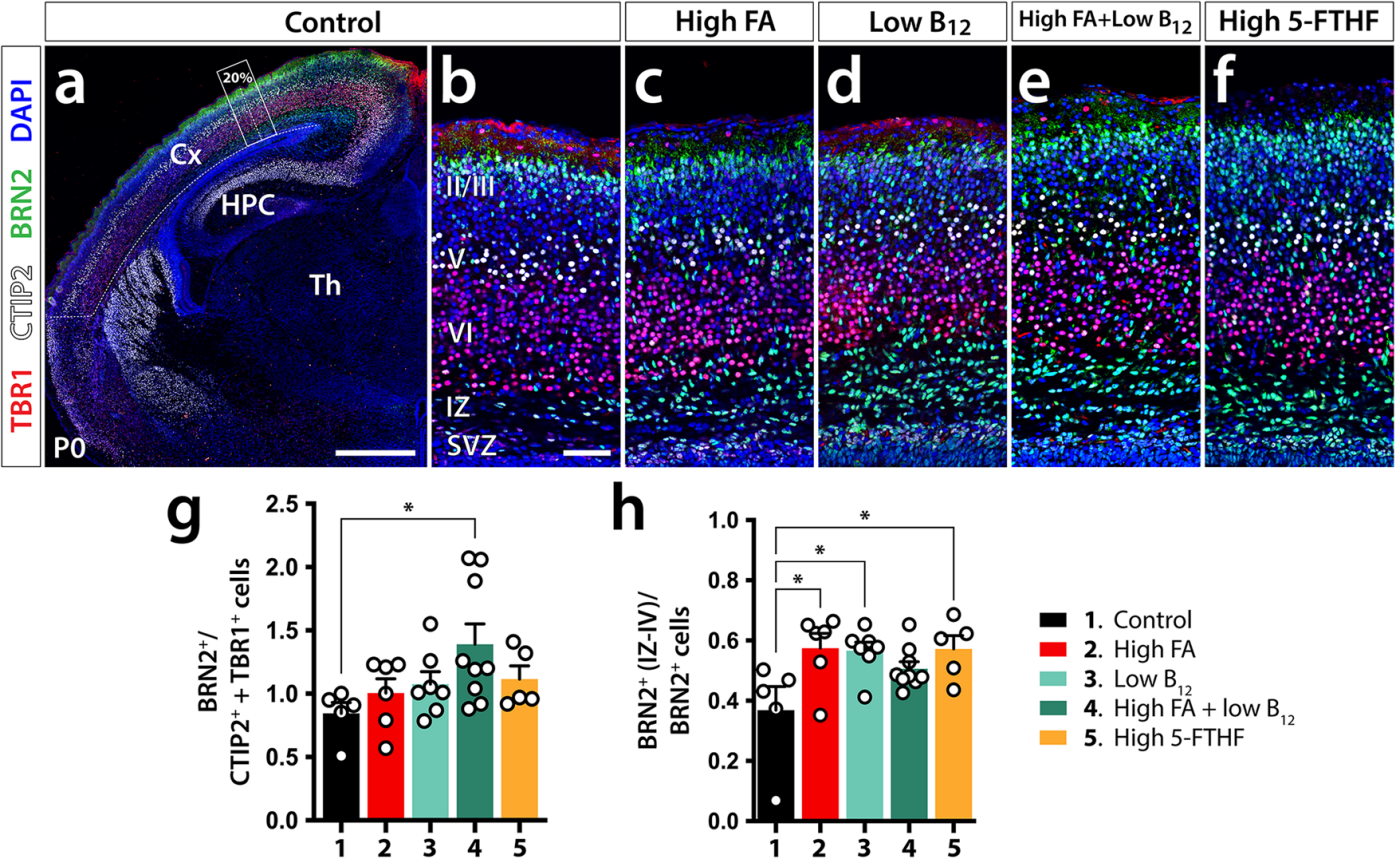
Laminar distribution and migration of cortical projection neurons at P0. **a** A control TBR1/BRN2/CTIP2 immunolabeled forebrain hemisection illustrates the position of sampled cortical segments. The dashed line indicates the measured neocortical length and the rectangular frame the sampled segment at 20% distance from the apex of the subventricular zone (SVZ). **b**–**f** Representative immunofluorescently labeled cortical segments of each dietary group used in the analysis of neuronal subtype distribution, respectively. **g** Bar diagram of the ratios of BRN2^+^ over CTIP2^+^ + TBR1^+^ neurons at the 20% distance mark shows a significant increase in group 4 compared with control. **h** Bar diagram of the ratios of BRN2^+^ outside layers II/III over all BRN2^+^ neurons show significant increases in high FA, low B_12,_ and high 5-FTHF test groups. Bar diagrams indicate means ± SEM and asterisks significant results by Dunnett’s test; *p ≤ 0.05. Cx cortex, HPC hippocampus, IZ intermediate zone, Th thalamus, Scale bar in **a** is 500 μm and in **b** 50 μm.

Informed by our earlier findings that recognized shifts in the distribution of deep layer vs. upper layer excitatory neurons^36^, we focused our initial analysis on assessing the relative distributions of cortical projection neurons. To identify excitatory neurons of deep layers V and VI, we used CTIP2 and TBR1 immunofluorescent analysis, respectively^49^. To distinguish upper layer neurons of layers II and III, we applied BRN2 immunolabeling^49^. To sample, we cut coronal sections and measured neocortical length along the lateral ventricle from the apex of the subventricular zone (SVZ) to the boundary with the entorhinal cortex and centered our analysis on dorsomedial neocortical aspects at a 20% distance from SVZ apex. Cell counts were performed in segments stretching 100 μm at P0 or 250 μm at P21 in either direction of this reference point. No overt alterations in layer organization between test groups were observed at either stage (Fig. 1a–f). To identify more subtle deviations in projection neuron distributions, we counted cells of different neuronal subtypes and established ratios of upper layer (II/III) over deep layer neurons (V/VI) as a proxy expression of potential cortical reorganization. Performing this analysis at P0, we recorded a significant increase in the ratio of analyzed cell types in group 4 only (high FA/low B_12_) compared with control (control: 0.844 ± 0.087; group 4: 1.39 ± 0.16, *p* = 0.0219) (Fig. 1g).

Comparing the total number of labeled cell types revealed that in group 4 only BRN2^+^ neurons were significantly increased compared with controls (control: 217 ± 21; group 4: 366 ± 46, *p* = 0.0322) (Supplementary Fig. 1). Furthermore, we noticed that the spatial distribution of late-born BRN2^+^ neurons appeared to differ in most test groups compared with control brains in such a way that a greater proportion of these cells apparently had not concluded their migration to their ultimate upper homing layers (II/III) with many of them still present in the intermediate zone (Fig. 1b–f). We proceeded to quantify this effect by establishing ratios of BRN2^+^ neurons apical to layers II/III over all BRN2^+^ neurons. Our analysis revealed significant increases of BRN2^+^ cells in deep layers of groups 2, 3, and 5 (high FA, low B_12_, and high 5-FTHF respectively) compared with controls suggesting a possible delay in birth and/or migration of late-born upper layer neurons (control: 0.37 ± 0.078; group 2: 0.57 ± 0.049, *p* = 0.0116; group 3: 0.57 ± 0.028, *p* = 0.0125; group 5: 0.57 ± 0.044, *p* = 0.0177). While group 4 also showed increases in deep layer located BRN2^+^ cells, this trend was not significant compared with controls (0.51 ± 0.023, *p* = 0.0836) (Fig. 1h).

### No cortical laminar aberrations in test groups at P21

Recognizing deviations in the distribution of BRN2^+^ neurons in newborn offspring that were manipulated in their FA/B_12_ supply, we sought to investigate whether these subtle changes may also lead to persistent changes in cortical neuronal composition. To answer this question, we analyzed P21 littermates of animals collected at P0, P21 being the timepoint of weaning at which lactation is switched to chow. At three weeks of age, cortical layer formation, including postnatal corrective mechanisms designed to eliminate inappropriately circuit-integrated neurons, is mostly completed. Brains were processed as described above and once again we used BRN2 as an upper layer marker (layers II, III, and V), but switched to CTIP2 to label deep layer neurons, as at this stage, CTIP2 more reliably marks excitatory neurons of both layers V and VI.

**Fig. 2:**
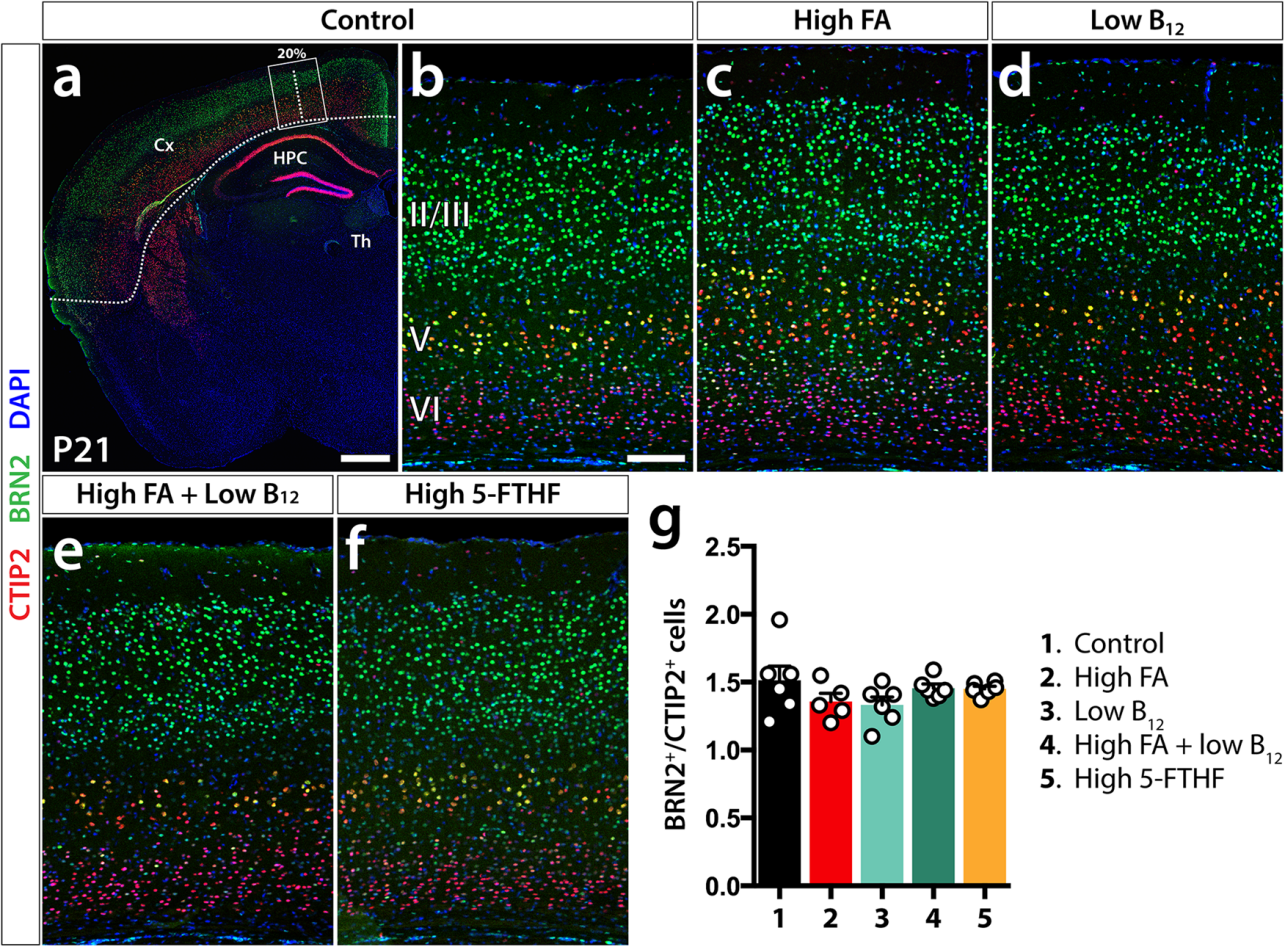
Laminar distribution of cortical projection neurons at P21. **a** Control BRN2/CTIP2 immunolabeled forebrain hemisection providing overview of sampled cortical segments. The dashed line indicates total neocortical length and the rectangular frame the sampled segment. **b***–***f** Representative cortical segments of dietary groups used in immunofluorescent analysis of upper and deep cortical layer excitatory neurons using BRN2 (layers II, III, and V) and CTIP2 (layer VI, V) respectively. **g** By establishing the ratios of upper vs. deep layer neurons at 20% neocortical distance from the cortical midline, no significant differences can be observed between dietary test groups and controls as the bar diagram of means ± SEM illustrates (Dunnett’s test). Scale bar in **a** is 500 μm and in **b** 100 μm.

At P21, we saw no significant differences in the numerical representation of upper vs. deep layer neurons between any of the experimental groups and controls. Specifically, control animals showed a mean ratio of BRN2^+^ (II, III)/CTIP2^+^ (V, VI) cells of 1.51 ± 0.1 while high FA (1.36 ± 0.06), low B_12_ (1.33 ± 0.06), high FA/low B_12_ (1.46 ± 0.03), and high 5-FTHF (1.45 ± 0.02) showed comparable and not significantly different values (Fig. 2). Cell density of either one of the assessed populations was also not disturbed and neither did we see significant changes in overall cortical thickness or thickness of BRN2 labeled layers II/III and CTIP2-labeled layers V/VI (Supplementary Fig. 2).

Considering that at P0 BRN2^+^ cells were significantly increased in group 4 compared with controls, but not so at P21, we sought to examine whether cell death contributed to the numerical development of BRN2^+^ neurons across time. To label apoptotic cells, we performed TUNEL assays at P0 and counted labeled cells across sections of entire cortical hemispheres. Comparing the number of TUNEL^+^ cells in the high FA/low B_12_ test group with controls, we recognized a significant increase of apoptosis in group 4 test animals, consistent with the idea of increased elimination of excess cells (control: 89 ± 7; group 4: 150 ± 15, *p* < 0.0066) (Supplementary Fig. 3a–c). To further confirm that apoptosis disproportionality affects BRN2^+^ cells, we performed at P0 CASP3 immunostaining in conjunction with BRN2 and TBR1 labeling. Comparing control to group 4 brains, we identified a significant increase in the ratio of CASP3^+^/BRN2^+^ cells over CASP3^+^/TBR1^+^ cells confirming a disproportionate loss of BRN2 neurons by apoptosis (control: 1.27 ± 0.54; group 4: 6.5 ± 1.1, *p* = 0.0079) (Supplementary Fig. 3d–f).

### FA excess or B_12_ deficiency diminishes dendritic complexity of cortical projections neurons

**Fig. 3:**
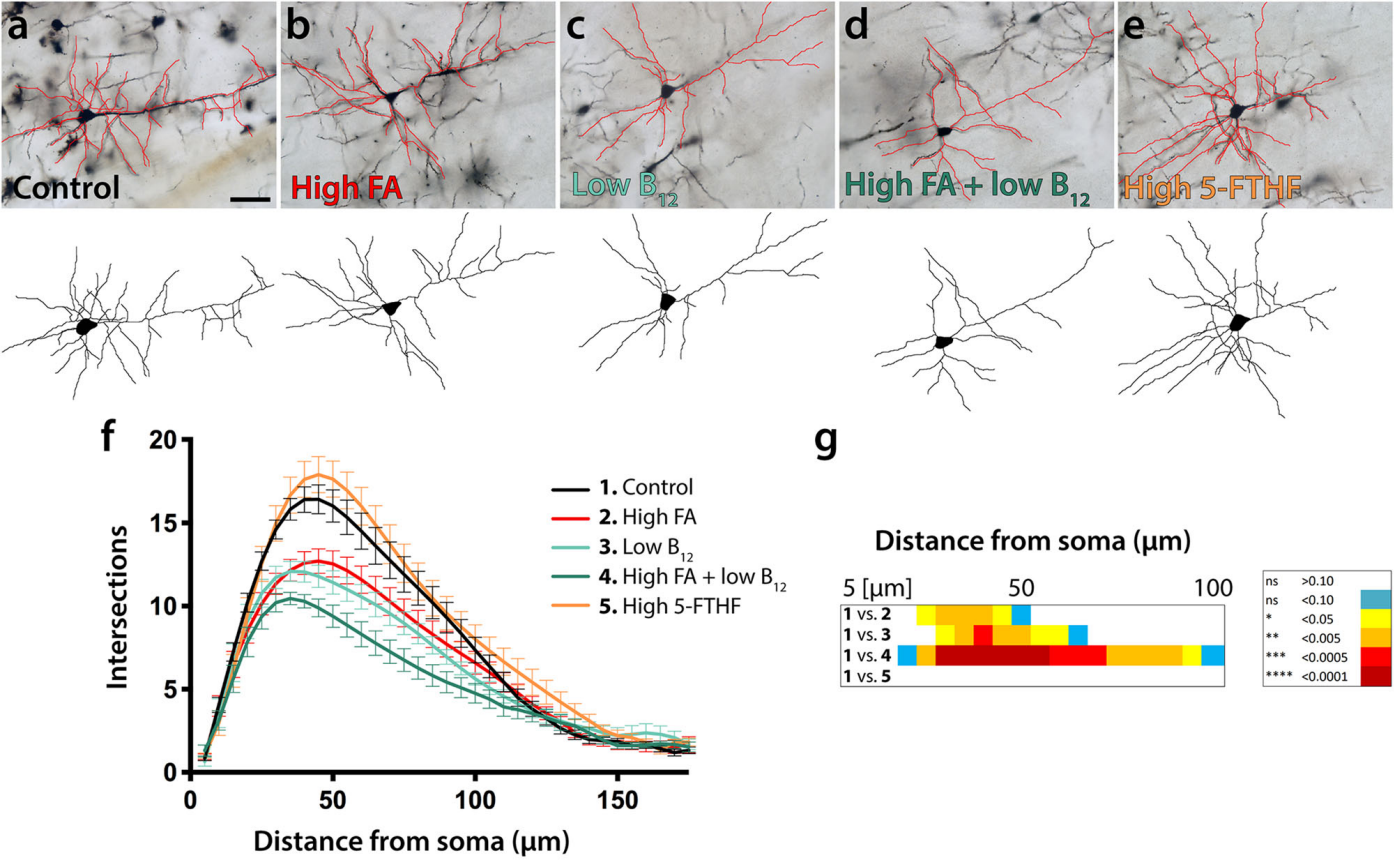
Dendritic arborization of deep layer pyramidal cells. **a**–**e** Representative micrographs of Golgi-stained deep layer projection neurons and tracings thereof illustrate reduced dendritic arborization in FA excess (**b**) and B_12_ deficiency (**c**, **d**) compared with control neurons. In contrast, excess 5-FTHF (**e**) does not show deviations compared with control. **f**, **g** Sholl profiles and statistical comparisons by ANOVA confirm significant differences between FA/B_12_ test groups and controls. The heatmap in (**g**) visualizes significance calculations between groups confirming significantly reduced complexity in groups 2–4. The legend on the right decodes *p*-value color associations. Scale bar is 50 μm.

To examine the subcellular and, by extension, physiological consequences of the cytoarchitectural changes that we observed in high FA and/or low B_12_ groups, we sparsely labeled cells and analyzed dendritic complexity of neurons using a modified Golgi-Cox staining protocol [FD Rapid GolgiStain kit (FD Neurotechnologies, Columbia, MD)]. The approach was informed by earlier findings showing fewer dendritic arbors in deep layer cortical projections neurons in either FA deficiency or excess compared with controls^36^, and our assumption that even transient structural defects may have functional consequences at the circuit level regarding neuronal interconnectivity. The complexity of dendritic arbors can be a proxy for neuronal circuit complexity and significant changes may be associated with neuronal dysfunction^50–52^. Analysis was performed in juveniles at postnatal day 21 and dendritic arborization examined by performing Sholl analysis. We focused on neurons of the dorsomedial somatosensory cortex at bregma −1.0 to −2.3 mm, the cortical area most affected by cytoarchitectural changes. First, we investigated deep layer projection neurons, observing reduced complexity in FA excess and both B_12_ deficient groups compared with controls (Fig. 3). In affected groups, significant differences were observed at distances from 25–100 μm from the soma with the number of peak intersections being diminished to 12.71 ± 0.74 in high FA (group 2) and 12.08 ± 0.61 in low B_12_ (group 3), compared with control (group 1, 16.43 ± 0.88 intersections). With maximally 10.45 ± 0.38 intersections, arbor reduction was most pronounced in group 4 combining high FA with low B_12_, suggesting an additive effect induced by the imbalance of both nutrients. In contrast, animals of group 5 that had received a diet high in 5-FTHF did not show any deviations compared with controls (group 5, 17.91 ± 1.08).

**Fig. 4:**
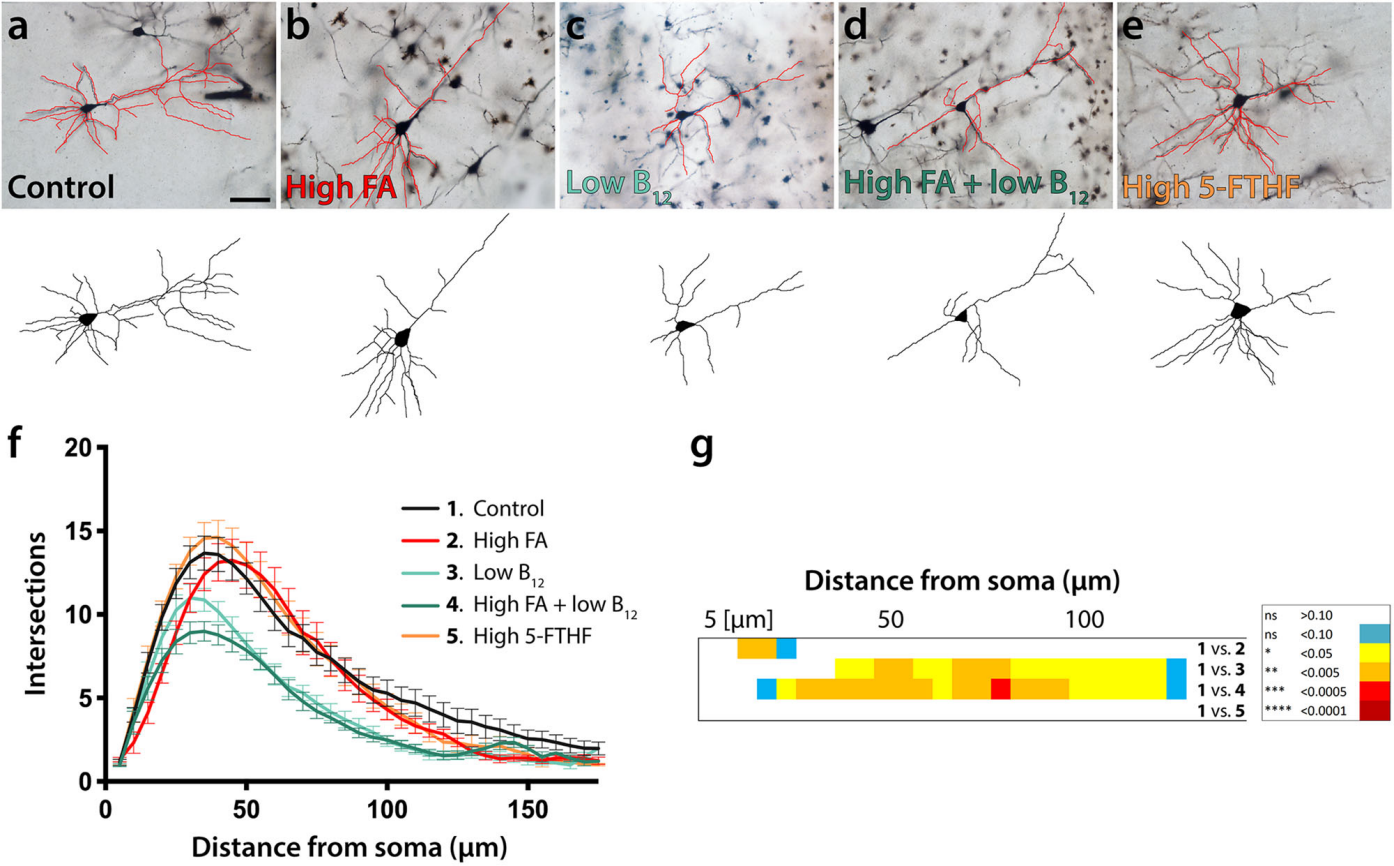
Dendritic arborization of upper layer pyramidal cells. **a**–**e** Representative micrographs of Golgi-stained upper layer projection neurons and respective tracings illustrate reduced dendritic arborization in both B_12_ deficient groups compared with control neurons (**c**, **d**). In contrast excess of FA or 5-FTHF does not show deviations compared with control (**e**). **f**, **g** Sholl profiles and statistical comparisons by ANOVA confirm significant differences between B_12_ test groups and controls. The heatmap in (**g**) visualizes significance calculations between groups confirming significantly reduced complexity in groups 3 and 4. The legend on the right decodes *p*-value color associations. Scale bar is 50 μm.

We expanded our analysis to also include upper layer projection neurons, a population of cells that at birth showed trends towards increase in all experimental groups and was significantly increased in group 4 (high FA/low B_12_) compared with control. Upper layer neurons of FA excess offspring (group 2) did not show fewer dendritic arbors (control: ~13.7 ± 1, group 2: ~13.2 ± 1.3) (Fig. 4). Similarly, group 5 offspring that had received a diet high in 5-FTHF did not deviate from controls with respect to arborization (group 5: ~14.6 ± 1). In contrast, offspring that had received low B_12_ with or without high FA, produced significantly diminished arbor complexity (group 3: ~11 ± 0.8, group 4: ~9 ± 0.6) (Fig. 4), following the paradigm of deep layer neurons.

### FA excess or B_12_ deficiency increases the density of dendritic spines of pyramidal cells

**Fig. 5:**
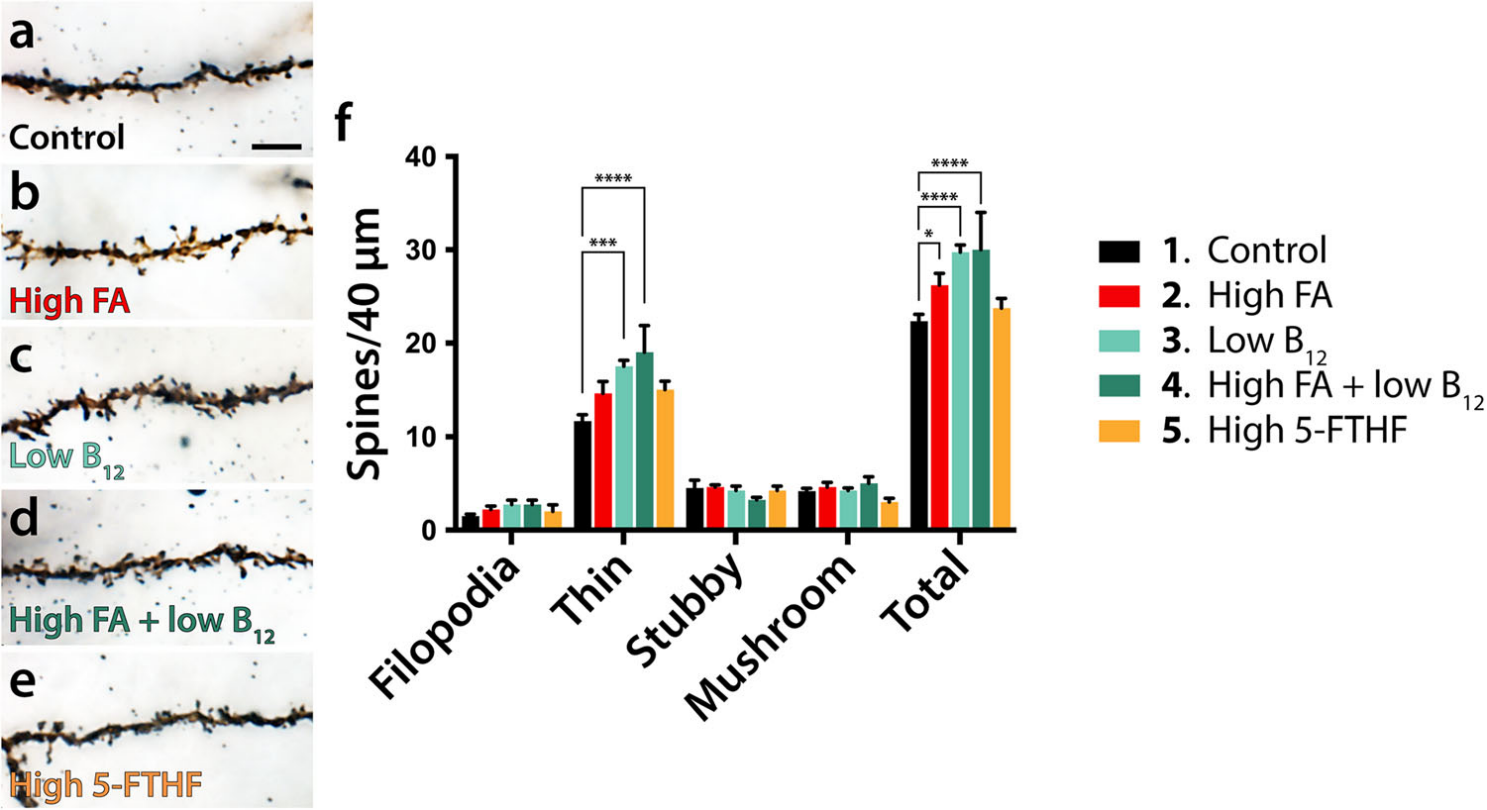
Dendritic spine analysis of deep layer pyramidal cells. **a**–**e** Representative maximum projection micrographs of Golgi-stained dendrites of deep layer projection neurons. **f** Bar diagram of means ± SEM illustrates the results of statistical analyses confirming significant increases (Dunnett’s test; *p ≤ 0.05, ***p ≤ 0.001, ****p ≤ 0.0001) in spine density between high FA or B_12_ deficient dietary groups (2, 3, and 4) and control with total spine effects mostly derived from increases in thin spines. Scale bar is 10 μm.

Deficiencies in dendritic arbor complexity may extend to informative changes in dendritic spine density and morphology. Multiple neurodevelopmental disorders have been associated with pathological alterations of both density and morphology of dendritic spines^53^. Using high-resolution z-stack images of Golgi impregnated projection neurons we distinguished between different subtypes of dendritic spines and individually determined their density in each dietary group (Fig. 5). We focused our analysis of group-dependent deviations on deep layer projection neurons, the neuronal population most consistently deviating from controls following FA/B_12_ manipulation. Our analysis revealed significant differences between dietary groups and controls. With respect to total spine density, high FA showed a significant increase (control: 22.33 ± 0.76, group 2: 26.20 ± 1.28, *p* = 0.0266) while both low B_12_ (group 3) and high FA/low B_12_ (group 4) showed significant increases in spine density compared with control (group 3: 29.75 ± 0.75, group 4: 30.00 ± 4.02, both *p* < 0.0001) (Fig. 5). In contrast, no significant effect on spine density was observed in the high 5-FTHF group compared with control. Increases in spine density of affected dietary groups was almost exclusively driven by an increase in thin (immature) spines where the same significant deviations were observed in groups 3 and 4 (control: 11.67 ± 0.67, group 3: 17.50 ± 0.65, group 4: 19.00 ± 2.89, *p* = 0.0008 and *p* < 0.0001 respectively) while no significant differences were observed for any other spine subtypes (Fig. 5).

### FA and/or B_12_ test groups display changes in folate cycle metabolic profiles

**Fig. 6:**
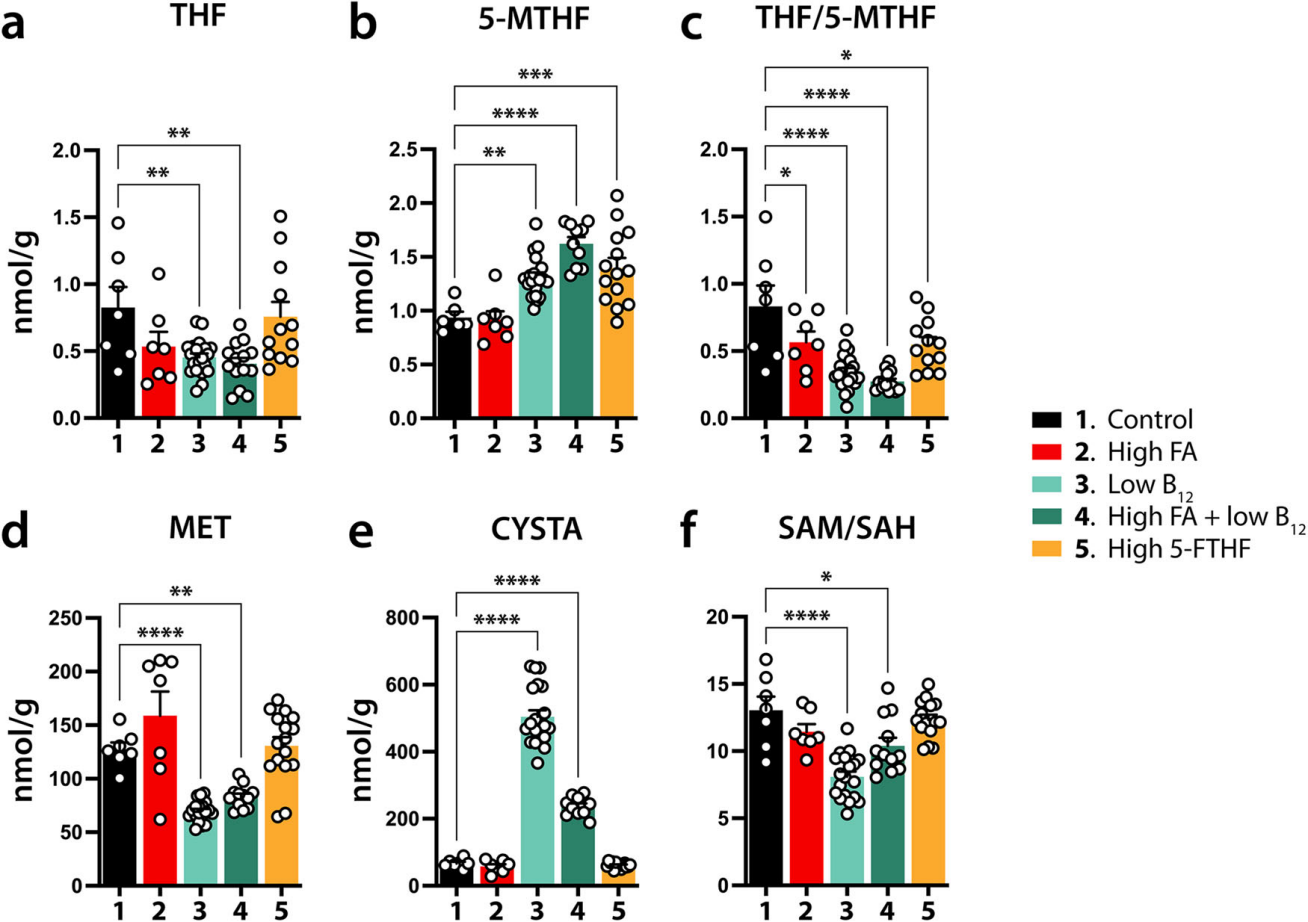
Folate and methionine cycle dysregulations in brains of newborn offspring. **a**–**f** Diagrams of means ± SEM depicting LC-MS/MS measured metabolite quantities and ratios in brains of newborn pups (P0) born to dams provided with control and experimental diets. Significant changes by Dunnett’s test are indicated by asterisks (*p ≤ 0.05, **p ≤ 0.01, ***p ≤ 0.001, ****p ≤ 0.0001). The low B_12_ (3), and high FA + low B_12_ (4) groups show the most consistent deviations from control animals in all metabolites and respective ratios shown. Notable are significant reductions in THF/5-MTHF ratios of all test groups (**c**) and SAM/SAH ratios of groups 3 and 4 (**f**), MET, and CYSTA values, as well THF/5-MTHF ratios. CYSTA cystathionine, 5-MTHF methyltetrahydrofolate, MET methionine, SAH S-Adenosyl-L-homocysteine, SAM S-adenosylmethionine, THF tetrahydrofolate.

**Fig. 7:**
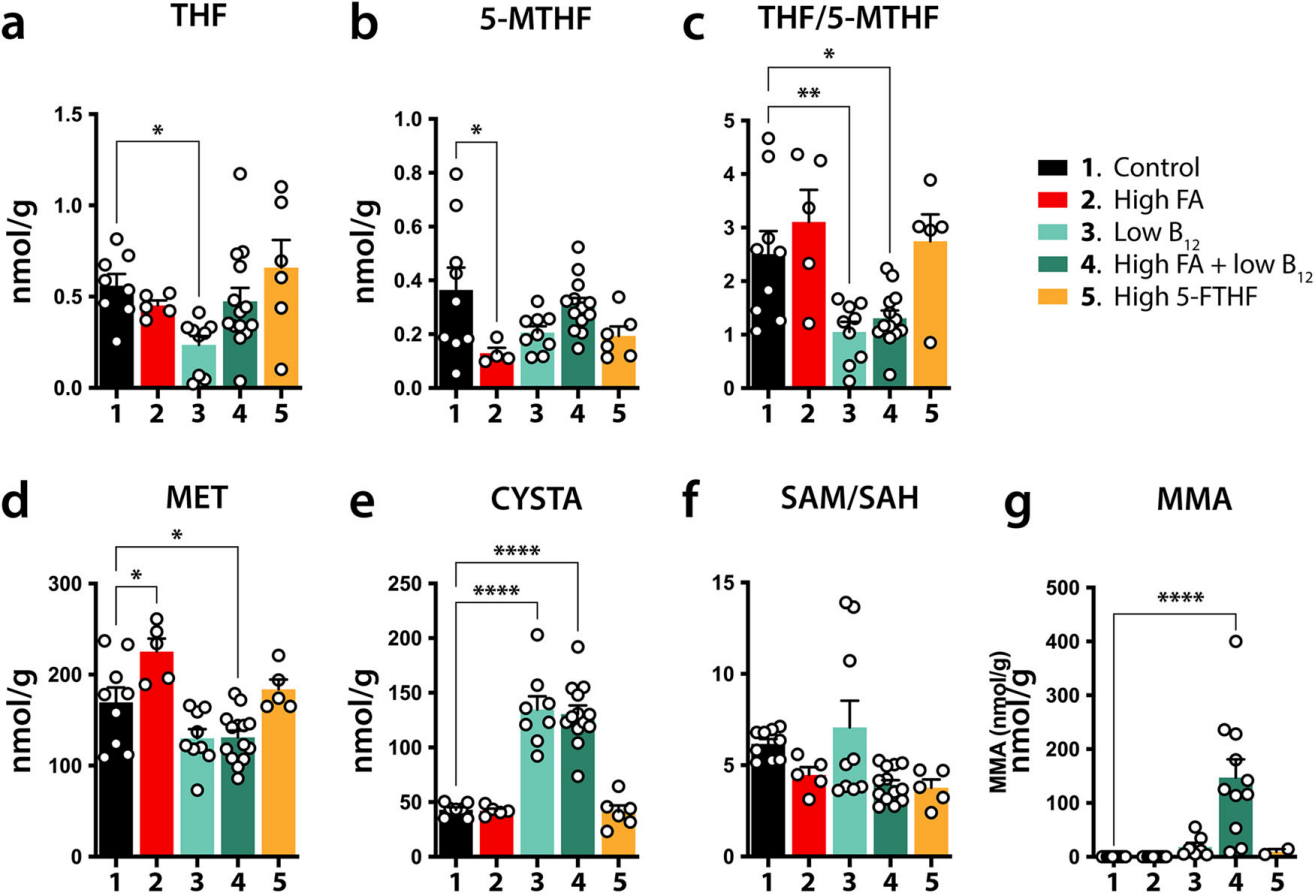
Folate and methionine cycle dysregulations in brains of P21 offspring. **a**–**g** Bar diagrams of means ± SEM representing LC-MS/MS measured metabolites and ratios in brains of three-week-old pups (P21) that were gestated under control and experimental conditions. Significant changes are indicated by asterisks (*p ≤ 0.05, **p ≤ 0.01, ****p ≤ 0.0001). Low B_12_ (3), and high FA + low B_12_ (4) show the most dramatic deviations from control animals in CYSTA values (**e**), as well THF/5-MTHF ratios (**c**). Notably, MMA shows a substantial increase in group 4 only, combining high FA with low B_12_ (**g**). CYSTA cystathionine, 5-MTHF methyltetrahydrofolate, MET methionine, MMA methylmalonic acid, SAH S-Adenosyl-L-homocysteine, SAM S-adenosylmethionine, THF tetrahydrofolate.

As we recognized consistent and significant morphological abnormalities in offspring under conditions of either FA excess, B12 deficiency, or a combination of both, we investigated whether the test diets administered to the dams produced changes in the profiles of folate species and one-carbon metabolites in the offspring. We performed these analyses in brain samples of both P0 and P21 pups. Specifically, we analyzed tetrahydrofolate (THF), methyltetrahydrofolate (5-MTHF), methionine (MET), cystathionine (CYSTA), S-adenosylmethionine (SAM), S-adenosyl-L-homocysteine (SAH), methylmalonic acid (MMA), choline (CHO), and betaine (BET). Applying mass spectrometric methods designed to measure absolute quantities, we identified significant differences for several metabolites in test groups compared with controls (Figs. 6 and 7). Unless expressed as ratios, values below are given as nmol/g tissue.

At P0, we noticed significant changes in all tested metabolites between controls and test groups (Fig. 6). Specifically, THF was significantly reduced in both B_12_ deficient groups (control: 0.825 ± 0.154; group 3: 0.454 ± 0.029, *p* = 0.0069; group 4: 0.406 ± 0.043, *p* = 0.0036) (Fig. 6a). Compared with controls, 5-MTHF showed significant increases in all groups except for the high FA group (control: 0.937 ± 0.054; group 3: 1.312 ± 0.043, *p* = 0.0045; group 4: 1.623 ± 0.063, *p* < 0.0001; group 4: 1.397 ± 0.092, *p* = 0.0008) (Fig. 6b). Observed deviations in THF and 5-MTHF levels also produced changes in THF/5-FTHF ratios, which saw significant decreases in all test groups compared with controls (control: 0.832 ± 0.156; group 2: 0.565 ± 0.080, *p* = 0.039; group 3: 0.347 ± 0.027, *p* < 0.0001; group 4: 0.274 ± 0.020, *p* < 0.0001; group 5: 0.549 ± 0.055, *p* = 0.0109) (Fig. 6c). The two B_12_ deficient groups showed significant decreases in MET (control: 127.6 ± 6.3; group 3: 69.6 ± 2.2, *p* < 0.0001; group 4: 83.6 ± 3.1, *p* = 0.0038) and significant increases in CYSTA (control: 66.7 ± 4.8; group 3: 504.2 ± 19.6, *p* < 0.0001; group 4: 236.8 ± 9.1, *p* < 0.0001). (Fig. 6d, e). Finally, SAM/SAH ratios were also significantly reduced in both B_12_ deficient groups (control: 13.03 ± 1.03; group 3: 8.084 ± 0.384, *p* < 0.0001; group 4: 10.39 ± 0.608, *p* = 0.0124) (Fig. 6f), while SAM was lower in both group 2 and 3 (control: 47.97 ± 2.32; group 2: 40.04 ± 3.35, *p* = 0.0408 ; group 3: 38.01 ± 1.51, *p* = 0.0009) and SAH was higher in group 3 (control: 3.76 ± 0.19; group 3: 4.79 ± 0.12, *p* = 0.0076) (Supplementary Fig. 4a, b). CHO, and BET were not affected in any of the experimental groups (Supplementary Fig. 4c, d).

At P21, particularly notable were significant reductions in THF/5-MTHF ratios in both B_12_ deficient groups (control: 2.504 ± 0.43; group 3: 1.053 ± 0.18, *p* = 0.0079; group 4: 1.307 ± 0.144, *p* = 0.0192) and increases in CYSTA (control: 43.03 ± 2.353 nmol/g; group 3: 134.8 ± 12.07 nmol/g, *p* < 0.0001; group 4: 130.6 ± 7.851, *p* < 0.0001) (Fig. 7). THF showed a significant reduction in the B_12_ deficient group only (control: 0.561 ± 0.064; group 3: 0.4516 ± 0.0274, *p* = 0.0268) and 5-MTHF a decrease in the FA excess group only (control: 0.364 ± 0.083; group 2: 0.129 ± 0.020, *p* = 0.0286). While SAM/SAH ratios were not affected in any of the experimental groups, SAM showed a significant decrease in group 4 (control: 29.02 ± 0.88; group 4: 25.18 ± 0.88, *p* = 0.0177) (Fig. 7 and Supplementary Fig. 5). MET was significantly increased in the high FA group (control: 169.7 ± 16.11; group 2: 225.4 ± 14.14, *p* < 0.0001) but significantly decreased in the high FA/low B12 group 4 (group 4: 131.2 ± 7.286, *p* = 0.0232). MMA showed a significant increase in group 4 only (control: <0.01 nmol/g; group 4: 147.1 ± 34.03, *p* < 0.0001) (Fig. 7). SAH, CHO, and BET did not significantly deviate in test groups compared with control (Supplementary Fig. 5).

## Discussion

Folate (B_9_) and vitamin B_12_ are essential micronutrients required for one-carbon metabolism on which cellular proliferation and differentiation depend^54,55^. Both vitamins have demonstrated associations with developmental growth and particularly neurodevelopment, as impaired folate transport to the brain causes cerebral folate deficiency^56^ and B_12_ deficiency can cause structural changes in the brain, as severe as atrophy^57^ and possibly a delay in myelination^58^ accompanied by early childhood neurological disorders^59,60^. Moreover, folate and B_12_ are interdependent, interacting within the same biochemical pathway concerned with the intersection of the methylation and folate cycles in the methionine synthase reaction. In this context, the combined effects of folate excess with B_12_ deficiency deserve particular consideration^61^.

The present study sought to explore the effects of high FA, low B_12_, and the interaction of FA/B_12_ imbalance in neurodevelopment. In the case of folate excess, we also investigated the question of whether the chemical form of folate in the diet has any influence on outcomes. Our findings suggest profound consequences of both FA excess and B_12_ deficiency on cortical neuronal development. Both dietary manipulations induced numerical imbalances in neocortical projection neuron generation that we identified at P0, while use of excess 5-FTHF as an alternative to high FA did not produce any overt deviations. In partial agreement with our previous work^36^, the results suggest a delay and/or temporal extension of prenatal neurogenesis leading to the generation of a surplus of late-born upper layer neurons in group 4 and trends in the same direction for all other groups. Confirmatory to this concept, in test groups of low B_12_ or high FA alone we recognized a delayed ascent of late-born BRN2^+^ neurons to the cortical plate. Interestingly, the numerical imbalances created by this neurogenic shift in group 4 appear to be substantially corrected postnatally, evident by P21. These corrections are likely based in the selective elimination of insufficiently circuit-integrated neurons that typically occurs within the first postnatal weeks, as our TUNEL analysis suggests. CASP3 analysis that included layer-specific neuronal markers further confirmed this assumption, as BRN2^+^ upper layer neurons were disproportionally more often CASP3^+^ compared with TBR1^+^ deep layer neurons.

Examining dendritic complexity of cortical neurons in experimental groups at P21 revealed decreased numbers of dendritic arbors compared with control offspring (except for 5-FTHF) suggesting enduring effects of imbalanced FA/B_12_ supply on neuronal morphology and circuitry outlasting postnatal numerical adjustments. We suspect that this finding is of particular importance in estimating the association between structural defects and neurological impairments, as the density of dendritic arbors can be a proxy for neuronal circuit complexity and significant reductions may result in neurological dysfunction^50–52^. In deep layer excitatory neurons of offspring subject to either high FA or low B_12_ diets, there is reduced dendritic arbor complexity compared with controls indicating striking parallels between the two dietary interventions. Diminished dendritic arborization is further exacerbated in group 4 that combines high FA with low B_12_ supply further supporting a synergistic effect of perturbations in these two micronutrients in influencing neurodevelopment. In addition, folate excess appears to influence the complexity of cortical connectivity only if supplied in the form of FA. In contrast the use of an equimolar administration of 5-FTHF, a stable reduced folate, had no discernable impact on arborization of deep layer cortical projection neurons. We surmise that this difference in behavior of FA and 5-FTHF relates to the fact that FA must first undergo reduction by the enzyme dihydrofolate reductase (DHFR). This enzyme reduces FA first to DHF and then to THF, the ultimately functional reduced form of folate. Under physiological conditions, DHF is the predominant substrate for DHFR and has a lower *K*_*m*_ for the enzyme than does FA. However, when FA is present in excess, the enzyme’s capacity to reduce DHF to THF becomes impaired and a state of functional folate deficiency results. Considering the significance of loss in dendritic arbor complexity for neuronal function, important insight comes also from the analysis of human neurodevelopmental syndromes and their relevant animal models. In Down, Rett, and fragile X syndromes multiple studies have reported diminished dendritic arbors [reviewed in^62–65^], suggesting a strong association between neuronal morphology and function.

Dendritic spine density also deviated in experimental groups compared with controls. We noticed significant or borderline significant increases in the total density of spines in all test groups, except for the high 5-FTFH group. The effect was mostly driven by increases in thin spine density that are considered to be immature and dynamic, produced during the formation of new memories and associated with synaptic plasticity^66,67^. This observation may suggest a period of extended cortical immaturity in our experimental groups, aligning well with our findings of prolonged generation and/or migration of upper layer neurons. Spine abnormalities have been found postmortem in multiple disorders that are often comorbid with or related to ASD and also in animal models for these disorders, including models for fragile X syndrome (FXS), Rett syndrome (RTT), and Angelman syndrome (AS)^53,68^. More specifically, increases in thin spine density have been noted in mice with astrocyte-restricted *Fmr1*^*-/y*^ deletion, a model for FXS^69^, and *Ube3a*^*m-/p+*^ mice, a model for AS^70^. Similarly, increases in thin spine density of cortical pyramidal neurons have also been noted in male Roman-High Avoidance rats (RHA-I), a genetic model presenting schizophrenia-relevant behavioral features, including impaired executive function^71^.

By measuring key metabolites involved in the folate cycle and methionine and transsulfuration pathways at P0 and P21, we observed that deviations in experimental groups were more pronounced in newborns compared with three-week old mice. A possible cause for this difference maybe that newborns have neither a developed gut microbiome nor access to chow yet that may further influence metabolite levels beyond conditions defined by maternal physiology.

While our findings regarding B_12_ deficiency may align well with the recognized importance of this micronutrient in brain development and function, the discussion on possible consequences of FA excess is more controversial. Considerable epidemiological research has investigated the association between FA intake and ASD rates and while some reports showed no association between prenatal FA supplementation and autism prevalence^21,22^ the majority of studies support a protective effect of FA on ASD prevalence^16–20^. A protective effect was also observed when FA intake was examined together with exposures to pesticides, air pollution, and anticonvulsants without clear mechanistic links between these exposures^72–74^. However, the binary design of these studies in which mothers were segregated into taking or not taking FA supplements without consideration of the actual amounts of consumed FA may obscure detrimental effects in those individuals in the highest quantiles of FA intake. In addition, actual folate blood levels or free FA are not reported in most of these studies. Some of the latest research in this area using data from the Boston Birth Cohort uncovered a positive association between maternal plasma folate levels and autism risk^25^. Autism incidence was greatest in children born to mothers with the highest maternal plasma folate levels, exceeding the cutoff suggested by the WHO (>45.3 nmol/L). Moreover, subsequent work showed that children with cord blood levels of unmetabolized FA (UMFA) in the highest, versus lowest quartile, had a greater risk for developing ASD^26^. Similarly, a Swedish study testing association of 62 maternal blood biomarkers during early pregnancy with later ASD diagnosis identified total folate as having the highest odds ratio at 1.7^75^. These results are supported by epidemiological investigations from the Rochester Epidemiological Project in Rochester, MN^23^ and earlier CDC data^24^. Beyond FA association with ASDs, the Spanish INMA study found that high amounts of supplemental FA (≥1000 μg/d) during pregnancy was associated with impaired neurocognitive development in children at 4–5 years of age^27^, intriguingly congruent with the findings that low doses of FA (≤400 μg/d), was also associated with attentional dysfunction in boys^28^. In summary, epidemiological studies have produced conflicting results with some suggesting protective effects while others support possible neurodevelopmental risks associated with excess FA exposure during pregnancy.

The novel findings in our mouse model lend support to the notion of a detrimental effect of even moderately excessive amounts of FA on prenatal cortical development that can be further aggravated by B_12_ deficiency. Two observations are of key importance: first, an apparent delay in the generation of late-born neurons, and second, a diminished complexity of excitatory neurons. B_12_ deficiency leads across most morphological and biochemical measures to greater deviations from the norm than FA excess. In contrast, replacing high amounts of FA by high amounts of 5-FTHF does not produce neurodevelopmental changes and milder folate cycle metabolic changes. Future animal studies as well as translational human studies may provide additional experimental insight in assessing neurodevelopmental risk associated with prenatal FA/B_12_ supply imbalances.

### Supplementary information

Supplementary Figures

Description of Additional Supplementary Files

Supplementary Data

Reporting Summary

## Data Availability

All data supporting the findings of this study are available in the main text or the supplementary materials. Numerical source data for graphs and charts can be found in the file named Supplementary Data.
