# Supplementary material for: Prenatal folic acid and vitamin B12 imbalance alter neuronal morphology and synaptic density in the mouse neocortex
Source: Commun Biol. 2023 Nov 8;6:1133. doi: 10.1038/s42003-023-05492-9 (PMC10632462; doi:10.1038/s42003-023-05492-9)
Supplement: Supplementary file 1 — Supplementary Figures [file 42003_2023_5492_MOESM1_ESM.pdf]

Supplementary Figures

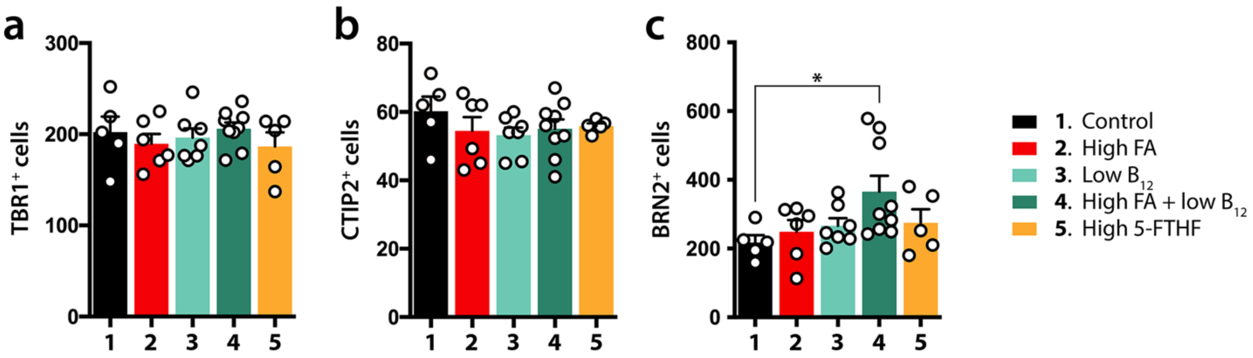

Supplementary Fig. 1. Total number of cortical projection neuron subtypes at P0. (*a*-*c*) Bar diagrams of means ± SEM depict quantifications and statistical comparisons (Dunnett's test) of test groups and control with respect to the numbers of TBR1⁺, CTIP2⁺, and BRN2⁺ cells per 200 μm cortical segment. A significant increase in BRN2⁺ neurons in group 4 only can be observed (*$p \leq 0.05$).

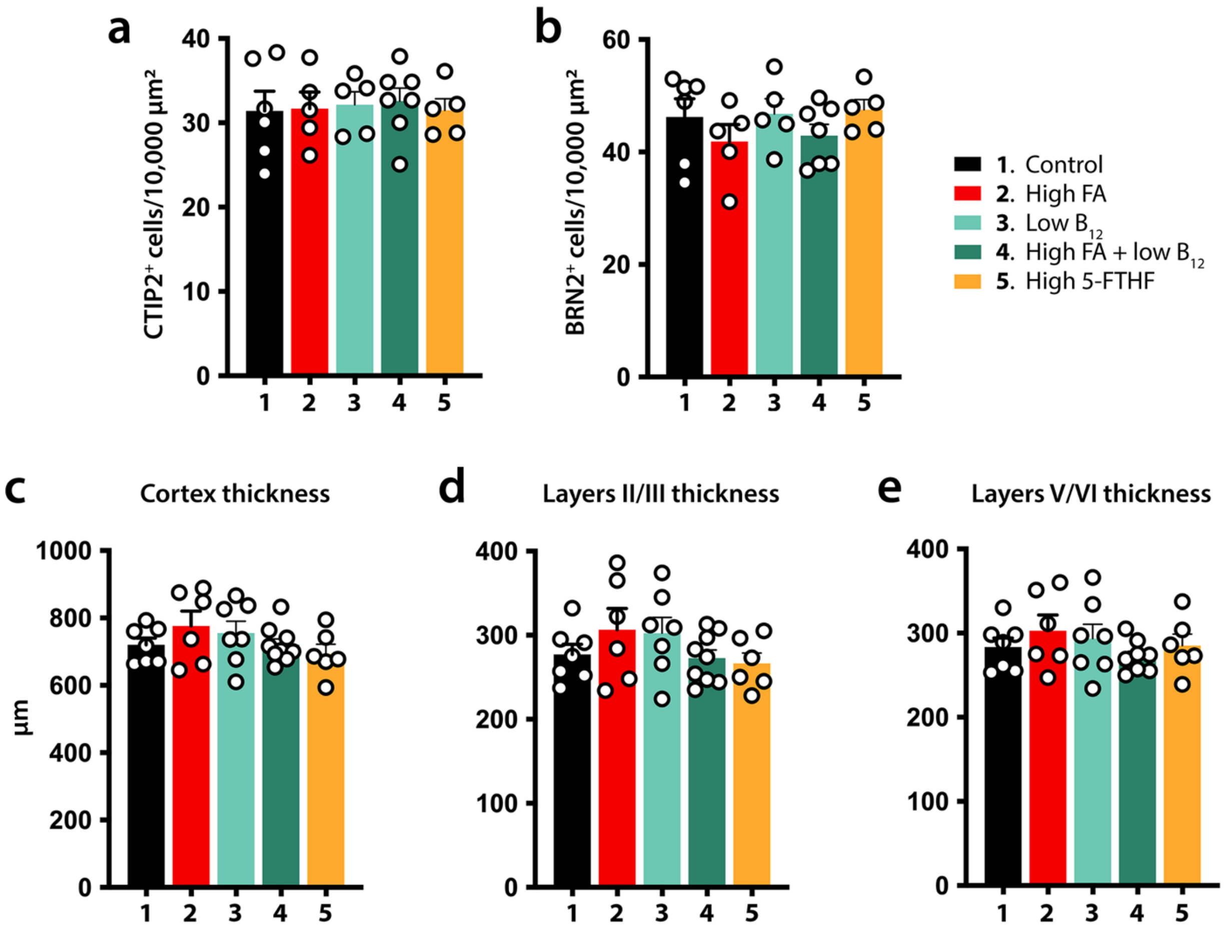

Supplementary Fig. 2. Neuronal cell density, cortical thickness, upper and deep layer thickness at P21. *(a-b)* Bar diagrams means ± SEM depict quantifications and statistical comparisons (Dunnett's test) of control and test groups with respect to the numbers of $CTIP2^+$, and $BRN2^+$ cells per 10,000 μm^2 of cortical area, respectively. No significant differences were noted after ANOVA testing. *(c-e)* Bar diagrams presenting total cortical, upper, and deep layer thicknesses respectively for control and experimental groups confirming no significant differences in any comparison.

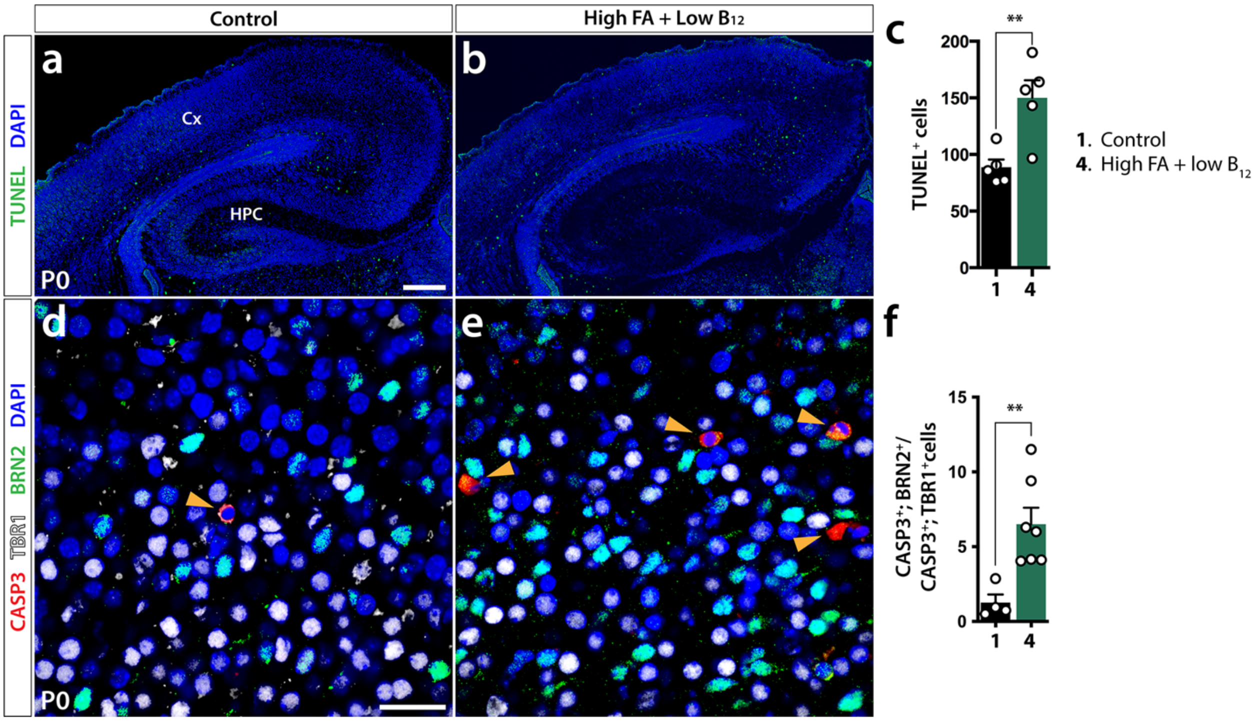

Supplementary Fig. 3. Total and cell type specific apoptotic rates in P0 cortex. *(a-c)* TUNEL analysis reveals a significant increase in TUNEL$^+$ cells in group 4 brains compared with controls at P0. *(d-f)* CASP3, TBR1, and BRN2 colabeling confirms a significant increase in the ratio of CASP3$^+$; BRN2$^+$ cells over CASP3$^+$; TBR1$^+$ cells in cortex of group 4 animals compared with controls at P0. Arrowheads in *D* and *E* point to CASP3$^+$; BRN2$^+$ double labeled cells. The bar diagrams (*c*, *f*) present means ± SEM and statistical comparisons by *t*-test (**$p \leq 0.01$). Cx: cortex, HPC: hippocampus. Scale bar in (*a*) is 200 μm and in (*d*) 20 μm.

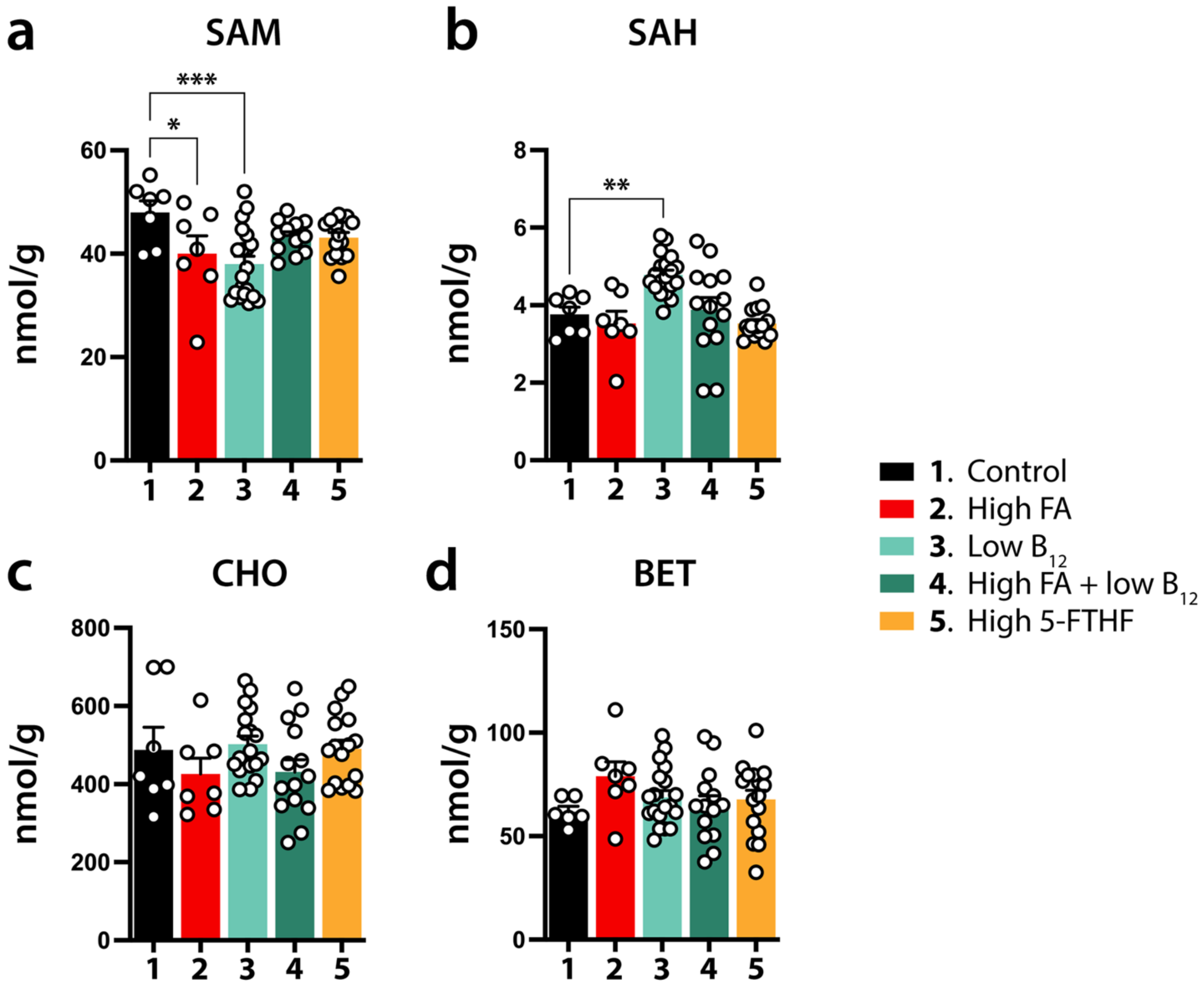

Supplementary Fig. 4. Folate and methionine cycle metabolite dysregulations in brains of P0 offspring. (*a-d*) Diagrams of means ± SEM showing LC-MS/MS determined metabolite quantities in brains of pups at P0 gestated under control and experimental conditions. Significant changes are indicated by asterisks (Dunnett's test; (*$p \leq 0.05$, **$p \leq 0.01$, ***$p \leq 0.001$). Compared with control, significant decreases in SAM were recorded in groups 2 (high FA) and 3 (low B_{12}) (*a*), while a significant increase was measured in group 3 (*b*). BET: betaine, CHO: choline, SAH: S-Adenosyl-L-homocysteine, SAM: S-adenosylmethionine.

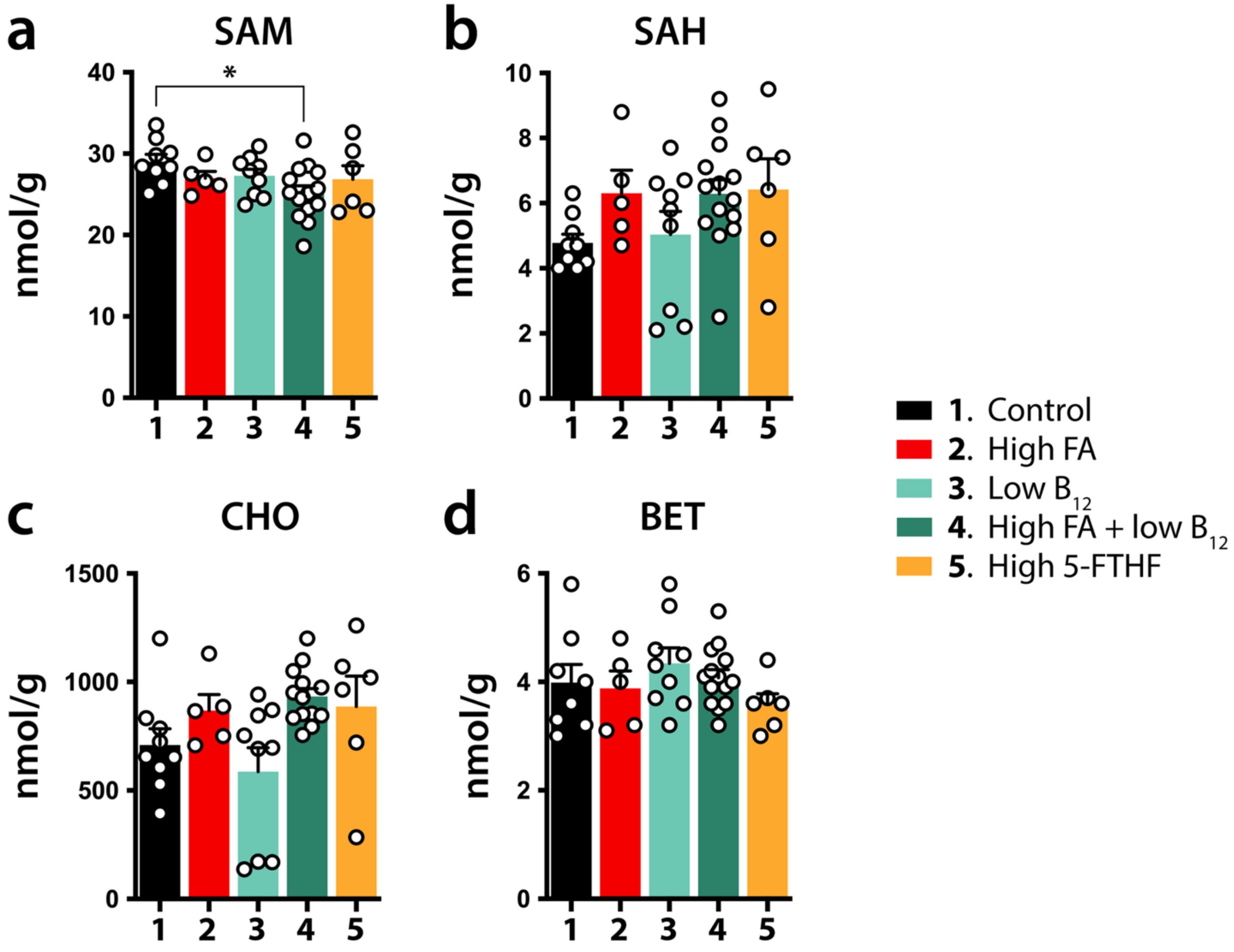

Supplementary Fig. 5. Folate and methionine cycle metabolite dysregulations in brains of P21 offspring. (*a-d*) Bar diagram of means ± SEM of LC-MS/MS measured metabolite quantities and ratios in brains of three-week-old pups (P21) gestated under control and experimental conditions. Significant changes are indicated by asterisks (Dunnett's test; (*$p \leq 0.05$). A significant decrease in SAM can be observed only in group 4 (high FA + low B_{12}) compared with control (*a*). BET: betaine, CHO: choline, SAH: S-Adenosyl-L-homocysteine, SAM: S-adenosylmethionine.
