# Supplementary material for: Prenatal folic acid and vitamin B12 imbalance alter neuronal morphology and synaptic density in the mouse neocortex
Source: Commun Biol. 2023 Nov 8;6:1133. doi: 10.1038/s42003-023-05492-9 (PMC10632462; doi:10.1038/s42003-023-05492-9)
Supplement: Supplementary file 2 — Description of Additional Supplementary Files [file 42003_2023_5492_MOESM2_ESM.pdf]

Description of Additional Supplementary Files

File name: Supplementary Data
Description: Numerical source data for graphs and charts.
